# Attenuated Semliki Forest virus for cancer treatment in dogs: safety assessment in two laboratory Beagles

**DOI:** 10.1186/s12917-015-0498-2

**Published:** 2015-07-28

**Authors:** Karoliina P. M. Autio, Janne J. Ruotsalainen, Marjukka O. Anttila, Minna Niittykoski, Matti Waris, Akseli Hemminki, Markus J. V. Vähä-Koskela, Ari E. Hinkkanen

**Affiliations:** Department of Equine and Small Animal Medicine, Faculty of Veterinary Medicine, University of Helsinki, P.O. Box 57, 00014 Helsinki, Finland; Cancer Gene Therapy Group, Department of Pathology and Transplantation Laboratory, Haartman Institute, University of Helsinki, P.O. Box 21, 00014 Helsinki, Finland; A. I. Virtanen Institute for Molecular Sciences, Biotechnology and Molecular Medicine, University of Eastern Finland, P.O. Box 1627, 70211 Kuopio, Finland; Finnish Food Safety Authority Evira, Pathology Unit, Mustialankatu 3, 00790 Helsinki, Finland; Department of Virology, University of Turku, 20014 Turku, Finland; Institute of Biotechnology, University of Helsinki, P.O. Box 56, 00014 Helsinki, Finland

**Keywords:** Oncolytic virus, Semliki Forest virus, Canine, Cancer

## Abstract

**Background:**

Dogs suffer from spontaneous tumors which may be amenable to therapies developed for human cancer patients, and dogs may serve as large-animal cancer models. A non-pathogenic Semliki Forest virus vector VA7-EGFP previously showed promise in targeting human tumor xenografts in mice, but the oncolytic capacity of the virus in canine cancer cells and the safety of the virus in higher mammals such as dogs, are not known. We therefore assessed the oncolytic potency of VA7-EGFP against canine cancer cells by infectivity and viability assays in two dog solid tumor cell lines. Furthermore we performed a 3-week safety study in two adult Beagles which received a single intravenous injection of ~2 × 10^5^ plaque forming units of parental A7(74) strain.

**Results:**

VA7-EGFP was able to replicate in and kill both canine cancer cell lines tested. No adverse events were observed in either of the two virus-injected adult Beagles and no infective virus could be recovered from any of the biological samples collected over the course of the study. Neutralizing antibodies to Semliki Forest virus became detectable in the dogs at 5 days post infection and remained elevated until study termination.

**Conclusions:**

Based on these results, testing of the oncolytic potential of attenuated Semliki Forest virus in canine cancer patients appears feasible.

**Electronic supplementary material:**

The online version of this article (doi:10.1186/s12917-015-0498-2) contains supplementary material, which is available to authorized users.

## Background

Canine tumors resemble human malignancies at cellular, phenotypic, and genomic levels, and dogs may serve as a more realistic cancer model system since their larger body size is more amenable to different surgical techniques and enables better evaluation of biodistribution of drugs and biotherapeutics than laboratory rodent models [1–3]. Because of the unmet therapeutic need in treatment of domestic animal neoplasms and the prospect of using pet cancer patients to narrow the gap to human clinical translation, the development of novel biological therapies to address canine malignancies is justifiable. Such therapies include oncolytic viruses, many of which are capable of infecting also non-human cells [4, 5]. Oncolytic viruses replicate preferentially in tumor cells and may potentially spread to distant metastases via the circulation. In addition, virus infection triggers inflammation which under optimal circumstances can break tumor immunosuppression and evoke anti-tumor immune responses [5, 6].

Alphaviruses are enveloped, single-stranded, positive sense RNA viruses of the family *Togaviridae,* and some of them have shown promising oncolytic potential in preclinical model systems [5, 7]. As animal viruses, alphaviruses may prove ideal candidates for development oncolytic virus therapies for dogs. Moreover, while most alphavirus strains show neurotropism allowing these viruses to pass the blood–brain-barrier to infect cells of the central nervous system (CNS), oncolytic alphaviruses based on nonvirulent strains remain apathogenic and are spontaneously cleared by the immune system [8].

In previous studies, oncolytic alphavirus VA7, which is based on an attenuated A7(74) strain of Semliki Forest virus (SFV), was capable of eradicating human brain tumors and melanoma xenografts in immunocompromised mice following just a single intravenous injection [9, 10], while in other occasions intratumoral administration route was proven superior [11–13]. Oncolytic capacity of attenuated SFV and also other alphaviruses is dependent on the lack of type I interferon responses in the tumor cells [14, 15]. However, little is known about SFV infections in dogs. In an early study, virulent prototype SFV strain caused neurologic signs of infection in puppies when administered intraperitoneally and intracerebrally but was asymptomatic upon intranasal, intradermal, or intracardial exposure [16]. The oncolytic capacity of SFV or any other alphavirus has not been investigated in canine cancer cells.

We hypothesized that attenuated SFV can infect and kill canine cancer cells and that it can be safely given to dogs intravenously. To test our hypothesis, we infected, as a proof-of-concept, two canine osteosarcoma cell lines (Abrams and D17) *in vitro* with SFV vector VA7-EGFP and assessed virus replication and cell killing. In addition, we performed a minimal safety study in non-tumor bearing adult Beagles using ~2 × 10^5^ plaque forming units (PFU) of the unmodified parental virus SFV A7(74) given via intravenous infusion. Our results show, on one hand, that dog tumor cells are able to support replication of oncolytic SFV, and on the other hand, that a limited inoculum of virus does not elicit side-effects in healthy adult dogs. These results pave the way for clinical translation of attenuated SFV in canine cancer patients.

## Results

### Cultured canine cells are permissive to SFV

**Fig. 1:**
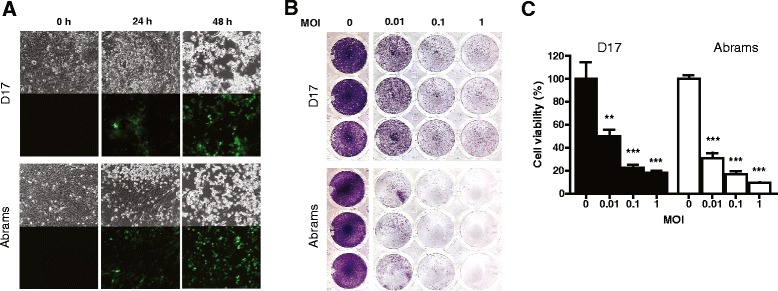
VA7-EGFP infects and kills the canine osteosarcoma cell lines Abrams and D17. Twenty-five thousand cells were plated on 48-well plate and infected next day with multiplicity of infection (MOI) 0.01, 0.1, or 1. The experiment was carried out as three replicates with identical results (one replica per sample type presented). **a** Infection with attenuated SFV vector VA7-EGFP was productive in both Abrams and D17 cells, as demonstrated by progressive increase in virus-expressed green fluorescent protein over time (lower panels, MOI 0.1), concomitant with virus-induced cytopathic effects seen in the corresponding phase contrast micrographs (upper panels). **b** Infection resulted in cell loss, shown by crystal violet staining at 48 h post infection (p.i.), where absence of staining indicates cell loss. **c** Quantification of acid-solubilized crystal violet from experimental wells in (**b**) demonstrated that oncolysis by SFV was virus dose-dependent, and that Abrams cells displayed slightly greater susceptibility to oncolysis with all MOIs tested. One asterisk represents *P* = 0.0053 and two *P* ≤ 0.0008, respectively. Values are shown in means and error bars indicate standard deviation

We first tested the oncolytic capacity of attenuated SFV in canine cancer cells by infecting two canine osteosarcoma cell lines, Abrams and D17, at various plaque-forming unit (PFU) to cell ratios (MOI = multiplicity of infection) with our reporter virus VA7-EGFP. Infection was productive in both cell lines, as seen by progressive increase in virus-expressed fluorescent reporter protein, concomitant with progressive cytopathic effects (Fig. 1a) and cell loss (Fig. 1b). Oncolysis was more pronounced in Abrams than in D17 cells at 48 h (Abrams: MOIs 0 vs 0.01, 0,1 and 1, *P* < 0.001 in all and D17: *P* = 0.0053, 0.008 and 0007, respectively) (Fig. 1b,c). The rate of oncolysis was comparable to sensitive human glioma cells [10].

### Intravenous administration of SFV displayed good safety in adult Beagles

**Fig. 2:**
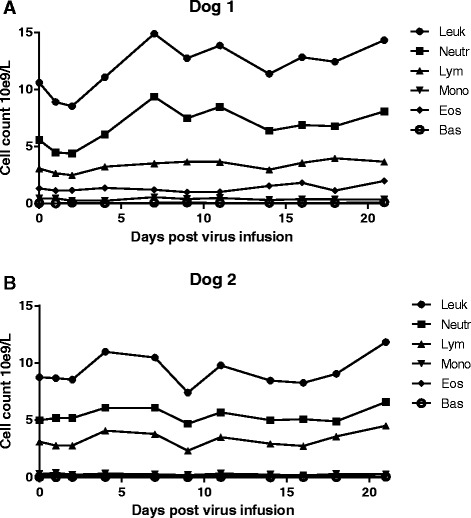
White blood cell count over time in dog 1 (**a**) and in dog 2 (**b**) after Semliki Forest virus infusion. SFV did not cause significant changes in any blood cell counts over the duration of the study. Leuk, leukocytes; Neutr, neutrophils; Mono, monocytes; Lym, lymphocytes; Eos, eosinophils; Bas, basophils

Two adult female laboratory Beagles were administered by intravenous infusion approximately 2 × 10^5^ PFU of non-recombinant parental SFV A7(74), accounting for a degree of virus inactivation in saline during animal preparation. We kept the injection dose relatively low to assess potential self-amplifying viremia, which is a hallmark of alphavirus infection in susceptible mammalian hosts [17, 18]. Neither dog showed any clinical signs of infection in any time post infusion. Total white blood cell and differential counts are presented in Fig. 2 and clinical chemistry in Table 1. These results revealed only a minor decrease in serum albumin concentration in dog 2 3 weeks after virus administration, while low blood glucose was detected in dog 1 1 week and in dog 2 3 weeks after virus administration. Further, dog 2 had slightly low sodium concentration three weeks after virus administration. In addition, both dogs had low serum bilirubin, protein, and cholesterol already at baseline compared to the reference values, which remained at similar levels during the study.

**Table 1:** Serum clinical chemistry of the Beagles receiving Semliki Forest virus

	Dog	1			Dog	2			
Day	0	7	14	21	0	7	14	21	Reference value
**ALP**	106	103	101	96	103	111	111	107	33–215 U/l
**ALAT**	34	41	37	34	26	29	31	33	18–77 U/l
**Albumin**	30.6	34.9	32.8	**29.9**	**26.2**	**27.3**	**27.4**	**26.3**	30–41 g/l
**Bilirubin**	**1.3**	**1.3**	**1.6**	**1.9**	**1.1**	**0.6**	**1.3**	**1.1**	2.5–8.5 μmol/l
**Phosphate**	1.34	1.46	1.29	1.44	1.51	1.76	1.27	1.28	0.93–2.25 mmol/l
**Glucose**	5.2	3.5	4.2	2.9	5.7	4.7	5.1	**3.6**	4.0–6.4 mmol/l
**Potassium**	4.8	4.6	4.6	4.5	5	4.9	5.1	5.3	4.2–5.4 mmol/l
**Sodium**	148	148	148	147	147	148	149	**144**	147–157 mmol/l
**Calcium**	2.58	2.88	2.7	2.65	2.58	2.56	2.54	2.51	2.3–3.0 mmol/l
**Cholesterol**	**3**	3.8	**3.3**	**3.1**	**2.5**	**2.6**	**2.4**	**2.1**	3.7–9.8 mmol/l
**Creatinine**	58	69	67	58	57	62	61	*55*	57–116 μmol/l
**Protein**	**49**	**57**	**54**	**48**	**45**	**50**	**49**	**45**	58–77 g/l
**Urea**	4.3	5.2	5.3	8.9	6.1	4.6	4.4	7.4	2.4–8.8 mmol/l

Bold numbers refer to values that are outside the reference range

### Infectious SFV was not detected in the sera, urine, or feces of the dogs

In order to determine the sensitivity of our virus detection assay in feces, a negative baseline sample (0.1 g) from each dog was spiked with 10^6^ PFU and diluted in DMEM. Virus recovery from these samples as shown by plaque assay was 6.25 × 10^4^ PFU/0.1 g (6.25 % recovery) and 3.75 × 10^5^/0.1 g PFU (37.5 % recovery) in dog 1 and dog 2, respectively. Thus, using the lower recovery rate, the smallest theoretical amount of feces giving at least one plaque in the assay was 400 PFU/g. Live virus could not be recovered from any of the analyzed serum, urine, or fecal samples. The theoretical detection limit for liquid samples was 25 PFU/ml.

### SFV infusion induced high levels of NAbs

Levels of neutralizing antibodies (NAbs) in serum samples collected before virus injection and 9 and 21 days post infusion were determined by plaque inhibition assay.

NAbs were not present at baseline, while NAb titers of 1250 on day 9 and 250 on day 21, respectively, could be detected in both dogs. Our results thus indicate that both dogs had obtained virus and mounted a humoral immune response in the absence of detectable viremia.

#### SFV administration did not induce histopathological changes

Necropsy and histopathology showed no specific findings associated with the virus administration. No virus antigen was detected in any of the organs analyzed.

## Discussion

Oncolytic viruses carry great potential as broadly active cancer therapeutics, as they simultaneously interfere with multiple cell survival signaling pathways and trigger inflammatory responses, which, in turn, may expose the tumors to the immune system. In the present study, we have assessed the potential of a promising oncolytic candidate, attenuated SFV, to target canine cancer. Like most human and rodent cancer cell lines in culture [5], also the two tested dog tumor cell lines were permissive to the virus (Fig. 1). Thus, SFV harbors intrinsic killing capacity against dog tumor cells, a prerequisite for the virus as a potential therapeutic for canine cancer.

While using a broader panel of dog tumor cells may reveal relative degrees of permissiveness to SFV, we have noticed that tumor sensitivity to lysis *in vitro* may not correlate to therapy efficacy *in vivo*, rendering extensive *in vitro* killing assays unwarranted [15, 19, 20]. Stromal cells and physical barriers contribute to restriction of virus spread *in vivo*, as infection triggers local inflammatory responses principally mediated by type I IFN to which the tumor cells may respond [21]. Paracrine cytokine signaling also serves to control alphavirus infection before further innate and adaptive immune responses have time to take place. However, in normal amplifying hosts, this signaling is sufficiently low to allow for systemic replication [22–24]. Indeed, the attenuated A7(74) used in our study typically induces in susceptible mammalian hosts an asymptomatic viremia of up to 10^8^ PFU/ml, lasting 1–4 days [17, 22, 25–27]. However, we did not detect any infectious virus in the blood, urine, or feces in the experimental dogs at any time *post* infusion, suggesting that healthy dog tissues are poorly permissive to SFV, resulting in absence of viremia and viral shedding. In theory, the used input dose of approximately 2 × 10^5^ PFU could have been too low to give rise to detectable viremia. While we cannot completely exclude this possibility, we do not believe it to be the case as very few viral particles are required for productive infection in susceptible host. Depending on the route of inoculation (and to some extent animal species), as little as 3–8 PFU constitute the 50 % protective dose for attenuated SFV strains against lethal SFV strain challenge, with productive replication and 100 % protection reached with 20 PFU administered peripherally [17, 22, 28]. Of note, rabbits, which are significantly larger than mice, still required only 20 PFU to receive 50 % protection against lethal challenge, and while protection against lethal virus challenge may occur in the absence of viremia, it does require productive replication, as defective or inactivated SFV elicits poor protective immune responses compared to live virus [29, 30]. Therefore, the avirulent SFV strain infection in normal animals is self-regulating and may present as viremia and/or neutralizing anti-SFV antibody response.

The neutralizing antibody response observed in both dogs in the present study indicated productive infection by the attenuated SFV, although no infectious virus was recovered from any blood samples or from the secretions. This is in line with the results from a recent study in healthy dogs injected with vesicular stomatitis virus, where infectious virus could not be detected in blood at any time *post* injection, but still all dogs developed antiviral antibodies from day 5 onward [31]. In a study with oncolytic adenovirus injected intravenously in healthy dogs infectious virus was not measured, but neutralizing antibodies increased already 4 days after virus administration [32]. So far, the only dog study in which infectious virus has been recovered after injection was with vaccinia virus, but the virus was recovered only from blood samples taken immediately after the intravenous administration [33]. Also in that study, neutralizing antibody responses were demonstrated 2 weeks after virus injection. The relevance of the anti-viral antibodies in oncolytic virotherapy remains incompletely understood, as their presence did not seem to correlate with treatment efficacy for example in patients receiving oncolytic vaccinia- [34, 35] or adenovirus [36–38]. However, the apparent difficulty to detect infectious viruses in dogs suggests rapid absorption of a number of different oncolytic viruses. This may have implications for intravenous delivery or systemic spread of oncolytic viruses in treatment of canine cancer patients. On the other hand, poor permissiveness of normal dog tissues to oncolytic viruses may be beneficial from a safety perspective.

Oncolytic adenovirus has so far showed excellent safety in tumor-bearing [39] and healthy dogs [32], likely owing in part to its restricted species tropism. Oncolytic vaccinia virus was reported to induce mild fever and an apparent epileptiformic seizure in healthy Beagles [33]. Several mild to moderate adverse events and one severe liver toxicity resulting in euthanasia were reported in healthy Beagle dogs receiving high-dose oncolytic vesicular stomatitis virus [31]. Unlike vesicular stomatitis virus, attenuated SFV can be safely injected intracranially in adult animals, where virus replication remains confined to mainly perivascular foci [11, 24, 40]. CD8+ T-cell-mediated elimination of the virus may in certain mouse strains be associated with demyelination [41–43], but such effects are transient and without apparent clinical symptoms [44]. Notably, we did not see any pathological changes or cellular infiltrates in the dog brains *post-mortem*.

In light of the poor capacity of SFV to replicate in dogs upon intravenous injection, clinical application of the virus in canine cancer patients would most likely entail intratumoral virus injections ensuring successful delivery of the virus into the tumor mass. This route has occasionally proven to be therapeutically superior over systemic administration even in susceptible hosts [11, 13]. Theoretically, SFV could amplify in tumors of canine cancer patients, disseminate into the blood and then be spread during the viremia by *Aedes* mosquitos endemic in the tropical areas [45]. Hence, viremia and shedding parameters should be established separately for canine cancer patients in the future.

## Conclusions

We report here the first proof-of-principle data for Semliki Forest virus as a candidate oncolytic agent in dogs. As our results demonstrate, live attenuated SFV VA7-EGFP was able to replicate and kill canine osteosarcoma cells *in vitro*, and parental A7(74) strain was safe causing no visible symptoms or distress in the laboratory Beagles. No virus could be detected in any of the organs, blood or secretions analyzed. Our findings support proceeding with SFV mediated oncolytic virotherapy to first stage clinical studies in canine cancer patients.

## Methods

### Virus and cell lines

Generation of the plaque-purified replication competent, attenuated A7(74) strain of SFV, kindly donated by Dr. R. E. Shope at the Yale Arbovirus Research Unit (Yale School of Medicine, CT, USA), has been described previously [17]. Generation of replication competent, attenuated VA7-EGFP expressing enhanced green fluorescent protein used in the *in vitro* studies has been also described earlier [8]. Viruses were amplified in BHK-21 baby hamster kidney cells (obtained from ATCC; Manassas, VA, USA) grown in high glucose (4.5 g/l) Dulbecco’s modified Eagles’s medium (DMEM; D6046, Sigma-Aldrich, St Louis, MO, USA) supplemented with 5 % fetal bovine serum (FCS), 2 mM L-glutamine, penicillin-streptomycin and 20 mM HEPES. The virus containing supernatant was collected 24 h *post* infection (p.i)., cell debris spinned down, and the supernatant sterile-filtered using 0.2 μm filter before freezing the virus in -70 °C in work aliquots.

Two canine osteosarcoma cell lines, Abrams (kindly donated by Dr. D. Vail, University of Wisconsin-Madison, USA,) and D17 (kindly donated by Dr. R. Alemany, Catalan Institute of Oncology, Barcelona, Spain) [46], were tested mycoplasma- free and grown in DMEM with 1 g/l glucose (D6046, Sigma Aldrich) supplemented with 1 % L-glutamine and 1 % penicillin-streptomycin. The culture medium was supplemented with 5 % FBS for Abrams and 10 % FBS for D17. Vero cells (obtained from ATCC) were grown in DMEM with 1 g/l glucose (D6046, Sigma Aldrich) supplemented with 25 mM HEPES, 1 % L-glutamine, 5 % FBS, and 1 % penicillin-streptomycin.

### Infection of canine cell lines

Abrams and D17 cells were plated on 48–well plates at a density of 25,000 cells/well. The next day, the cells were infected with VA7-EGFP at MOI of 0.01, 0.1, and 1. Growth medium was used as sham treatment and all samples were prepared as triplicate with identical results. Virus infection was monitored by fluorescence microscopy and cytopathic effect was evaluated by phase contrast microscopy (Zeiss Axio Observer Z1 microscope, Zeiss, Oberkochen, Germany) 24 and 48 h p.i. Finally, crystal violet staining was performed to reveal cytopathic effect at the final time point of 48 h p.i. and stained plates were imaged with digital camera. For quantification of cell viability, crystal violet from stained wells was dissolved in 250 μl 10 % acetic acid for 20 min in room temperature in a shaker. Samples from dissolved dye were transferred to 96-well plate and absorbance was measured at 595 nm wavelength with plate reader (VICTOR2, PerkinElmer, Waltham, MA, USA).

### Dogs

Two healthy adult female HsdRcc:DOBE Beagle dogs (Harlan Laboratories, Gannat, France) were infected with A7(74) strain of SFV. Both dogs were 1 year old retired breeding females. The dogs were housed together in standard conditions, fed twice daily, and given water *ad libitum*. The experiment was approved by the National Animal Experiment Board of the Regional State Administrative Agency of Southern Finland (ESAVI/3231/04.10.07/2013). Dog 1 weighed 6.7 kg and dog 2, 12.9 kg.

### Study design

Before virus administration, an intravenous catheter was placed into the cephalic vein and 10 ml of sterile saline was administered to ensure vein access. As SFV is pH-sensitive and could potentially suffer from inactivation in physiological saline which lacks buffering capacity, we chose to inject 1 million PFU of the virus in order to expose the dogs to a minimally infective dose (see Discussion). Virus was diluted in 50 ml of saline (0.9 % Sodium Chloride Irrigation, Baxter, Norfolk, UK) and kept on ice until given to the dogs via intravenous infusion over 15 min (200 ml/h). The infusion line ran through tempered water bath (37 °C) before entering the vein. After virus administration, the virus-saline bag and infusion line were flushed with 25 ml of saline at the same infusion rate to ensure that the dogs received the entire dose of virus. Altogether the infusions lasted for approximately 23 min.

The dogs were monitored for adverse events according to the Veterinary Cooperative Oncology Group - Common Terminology Criteria for Adverse Events (VCOG-CTCAE) v1.1 [47]. Attitude, respiratory rate, heart rate, color of mucous membranes, capillary refill time, rectal temperature, and blood pressure were monitored before virus administration, then 15 and 30 min, and 1, 2, 4, 8, 11, and 24 h after the initiation of virus infusion. In addition, the dogs were monitored for swelling, itching, and vomiting, which are the most common signs of hypersensitivity in this species. After 24 h, attitude and appetite were monitored twice daily, and weight, respiratory rate, color of mucous membranes, capillary refill time, and rectal temperature once daily. The dogs were euthanized 3 weeks after virus infusions, and had full necropsy.

### Sample collection

**Fig. 3:**
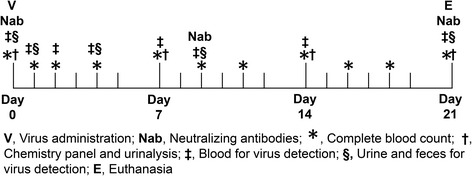
Study chart and sample collection for safety evaluation of Semliki Forest virus in two Beagle dogs. *V* Virus administration, *Nab* Neutralizing antibodies, * Complete blood count, † Chemistry panel and urinalysis, ‡ Blood for virus detection, § Urine and feces for virus detecttion, *E* Euthanasia

The sample collection schedule is shown in Fig. 3. Four milliliters of blood were collected into serum tubes for virus detection from jugular, cephalic, or saphenous vein before and 2, 4, and 22 h and 2, 4, 7, 9, 14, and 21 days after virus infusion. Urine (5 ml) was collected by cystocentesis and feces (10–15 g) were collected rectally. Serum was separated after 20 min incubation in room temperature by centrifuging 10 min 2400 g. It was then frozen first at -18 °C and transferred the same day into -80 °C until virus titer analysis. Urine and fecal samples were frozen and stored according to the same protocol.

One milliliter blood was collected for hematology before, and 1, 2, and 4 days after virus administration and then three times weekly. In addition, 6 ml of blood were collected into serum tube for clinical chemistry and the assessment of SFV antibody titers before virus infusion and then, 9–21 days after virus administration. Serum was separated as described earlier. Hematological parameters were determined by an automated analyzer (ADVIA 2120i Hematology System, Siemens Healthcare Diagnostics Inc., Tarrytown, NY, USA) and serum was assayed by a clinical chemistry analyzer (Konelab 30i, ThermoFisher Scientific, Vantaa, Finland). Urinalysis was done once a week by dipstic (Multistix ®10 SG, Siemens Healthcare Diagnostics Inc.) read with automated reading device (Clinitek Status, Siemens Healthcare Diagnostics Inc.) Specific gravity was determined by refractometry, and a standard volume (0.5 ml) of urine sediment was examined under a light microscope using 10× and 40× objectives after centrifugation (5 min 600 g). Urine protein-creatinine ratio was measured by a clinical chemistry analyzer (Konelab 30i). Samples for SFV antibody titer were frozen first at -18 °C and transferred the same day into -80 °C until analysis. Hematology, chemistry panel, and urinalysis were analyzed the day they were collected.

### Virus titration

Sera obtained from the dogs were titered for SFV A7(74) on Vero cells according to Ruotsalainen *et al.* [15]. Serial dilutions of samples were prepared in cold medium. Stock A7(74) virus was used as a positive assay control. In order to exclude the possibility that the virus had been inactivated during infusion procedure, we simulated the conditions of the virus administration protocol by keeping the A7(74) virus -containing sterile saline infusion bag on ice for 3 h 25 min. This was the maximum time from the virus infusion preparation, performed in a separate BLS2 facility, until the end of the virus infusion. We then keept the virus-saline mixture for 1 min in a 37 °C water bath, and finally collected and titered the viral solution by plaque assay in Vero cells. The protocol for virus titration from urine and feces samples was modified from Buonacurio *et al.* [48]. Urine samples were diluted 1:10 and 1:100 in DMEM with 1 g/l glucose and virus titration was performed on Vero cells as for the sera. Before virus titration from fecal samples 10× (w/v) of DMEM was mixed with the feces (0.1–0.2 g), and centrifuged at 3000 g for 10 min at 4 °C. The supernatant was sterile-filtered using 0.22 μm polyethersulfone membrane syringe filter (Syringe Filter, Porvair Science, Leatherhead, UK) and diluted in growth medium 1:10 and 1:100 for titration purposes. Recovery controls were prepared for fecal samples by adding 10^6^ PFU of SFV A7(74) per 0.1 g of baseline fecal samples of the two dogs diluted in 10× (w/v) amount of medium. The virus-feces-medium mixtures were then vortexed, sterile filtered, serial diluted (from 10^−1^ to 10^−6^) in growth medium, and titered as described above.

### Neutralizing antibodies

NAbs were measured from the serum samples collected on baseline, and 9 and 21 days after the virus infusion according to Ruotsalainen *et al.* [19]. Briefly, titering of NAbs was performed on Vero cells plated on 12-well plates. The serum samples were diluted 1/2, 1/5, 1/25, 1/125, 1/625 and 1/3125 in DMEM, and the dilutions mixed 1:1 (100 μl:100 μl) with A7(74) solution containing 50 PFU of virus. The mixture was incubated on ice for approximately 30 min before plaque titering on Vero cells seeded on 12-well plates as duplicates. Pure medium served as a negative control, and polyclonal rabbit anti-SFV antibody in DMEM as a positive control. Fluorescence was monitored by fluorescence microscope (Zeiss Axio Observer Z1 microscope). The neutralizing antibody titer was defined as the greatest dilution of virus completely preventing plaque formation.

### Necropsy

Necropsy and histopathological examination were performed by a board-certified pathologist (MOA). Representative samples of all selected organs (Additional file 1) were collected and fixed in 10 % buffered formalin. All samples were dehydrated, embedded in paraffin, sectioned, and stained with hematoxylin and eosin. In addition, tissue samples obtained from organs previously known to harbor SFV in mice [9, 49] (brain hippocampus, cortex, corpus callosum, cerebellum and pons; kidneys; liver; lymph nodes: mandibular and mesenteric; skeletal muscle; spleen; cervical spinal cord) were stained by polyclonal rabbit anti-SFV antibody using Vectastain ABC kit (Vector Laboratories, Burlingame, CA, USA) followed by hematoxylin counterstain. SFV envelope antigen-positive mouse brain was used as a control. Sections were examined under microscope via 40× and 10× objectives.

## Statistical analysis

Cell viability in percentages was counted from absorbance using formula: (A595(sample) / mean A595(uninfected sample)) × 100. The data was plotted and analyzed with GraphPad Prism (GraphPad Software, Inc. La Jolla, CA, USA). Statistical analysis was done using unpaired, two-tailed *t*-test and *P* < 0.01 was considered significant.

## Additional file

Additional file 1: 
**Tissue list for histopathology collection from the dogs receiving Semliki Forest virus (SFV).** (DOCX 14 kb)

## Abbreviations

DMEM: Dulbecco’s modified Eagles’s medium
EGFP: Enhanced green fluorescent protein
FBS: Fetal bovine serum
HEPES: 4-(2-hydroxyethyl)-1-piperazineethanesulfonic acid
MOI: Multiplicity of infection
NAbs: Neutralizing antibodies
p.i.: *post* infection
PFU: Plaque forming units
SFV: Semliki Forest virus
