# Supplementary material for: Attenuated Semliki Forest virus for cancer treatment in dogs: safety assessment in two laboratory Beagles
Source: BMC Vet Res. 2015 Jul 28;11:170. doi: 10.1186/s12917-015-0498-2 (PMC4515883; doi:10.1186/s12917-015-0498-2)
Supplement: Additional file 1: — Tissue list for histopathology collection from the dogs receiving Semliki Forest virus (SFV). (DOCX 14 kb) [file 12917_2015_498_MOESM1_ESM.docx]

**Additional file 1**

Tissue list for histopathology collection from the dogs receiving Semliki Forest virus (SFV).

| Adrenal glands (cortex and medulla) | Ovaries |
| --- | --- |
| Bone and joint (distal femur) | Pancreas |
| Bone marrow (rib) | Peripheral nerve (sciatic) |
| Brain* | Pituitary gland |
| Gut associated lymphoid tissue | Salivary gland (submandibular) |
| Heart | Skeletal muscle (thigh) |
| Jejunum | Skeletal muscle (diaphragm) |
| Kidneys | Spinal cord (cervical and lumbar) |
| Liver | Spleen |
| Lungs | Thymus |
| Lymph nodes, mandibular | Thyroid/Parathyroid glands |
| Lymph nodes, mesenteric | Uterus |
| Optic nerves | Vagina |

* hippocampus, cortex, corpus callosum, cerebellum and pons
